# Molecular imaging of tucatinib-induced cellular and TME changes in preclinical models of HER2 + breast cancer

**DOI:** 10.1007/s10549-026-07936-2

**Published:** 2026-03-13

**Authors:** Patrick N. Song, Ameer Mansur, Carlos A. Gallegos, Pragya Ghanate, Suzanne E. Lapi, Anna G. Sorace

**Affiliations:** 1 Department of Radiology, The University of Alabama at Birmingham, Birmingham, USA; 2 Graduate Biomedical Sciences, The University of Alabama at Birmingham, Birmingham, USA; 3 Department of Biomedical Engineering, The University of Alabama at Birmingham, Birmingham, USA; 4 O’Neal Comprehensive Cancer Center, The University of Alabama at Birmingham, Birmingham, USA; 5 Advanced Medical Imaging Research Division Radiology, Department of Radiology and Biomedical Engineering, The University of Alabama at Birmingham, 1670 University Blvd, Birmingham, Alabama, 35233 USA

**Keywords:** Positron emission tomography, PET, BT474, BCM3472, PET, FMISO, FLT, [^89^Zr]Zr-Pertuzumab, Small molecule inhibitor

## Abstract

**Introduction:**

Tucatinib, a small molecule HER2 inhibitor, was approved in inoperable or metastatic HER2 + breast cancer. As many patients have tumors in challenging surgical locations, there is a need for imaging metrics to characterize tucatinib response and microenvironment impact. Molecular imaging can be used to quantify dynamic molecular changes that precede tumor size alterations and can target proliferation (fluorothymidine, [^18^F]-FLT), hypoxia (fluoromisonidazole, [^18^F]-FMISO) and HER2 expression ([^89^Zr]-Pertuzumab) with positron emission tomography (PET) imaging. The goal of this study is to non-invasively characterize tucatinib response in HER2 + breast cancer and quantify microenvironment modulation with advanced PET imaging.

**Methods:**

Mice with HER2 + human cell line (BT474) or patient derived xenograft (BCM 3472) tumors were treated with 50 mg/kg tucatinib and enrolled into imaging cohorts: imaged with [^18^F]-FLT-PET on days 0, 3 and 7, [^18^F]-FMISO-PET on days 0, 3 and 7, or [^89^Zr]Zr-Pertuzumab-PET on days 0 and 14. Proliferation, hypoxia and HER2 expression were quantified with standardized uptake value. A Mann–Whitney U Test assessed significance between groups.

**Results:**

Tucatinib-treated human cell line and PDX tumors had significantly decreased hypoxia and proliferation relative to control tumors (p < 0.05). Tucatinib-treated BT474 tumors had significantly decreased HER2 expression (p < 0.05); however, no significant HER2 change was observed in BCM3472 tumors.

**Conclusion:**

Tucatinib significantly decreases intratumoral proliferation and hypoxia in both cell-line and patient-derived xenograft models of HER2 + breast cancer, which can be longitudinally quantified with PET imaging. Our data suggests molecular imaging may improve understanding and prediction of tucatinib response.

## Introduction

Breast cancer is a heterogeneous disease and is one of the most prevalent malignancies among women worldwide, affecting 1 in 8 women [1–3]. Approximately 20% of breast cancers are classified as HER2-positive (HER2 +), characterized by overexpression of the human epidermal growth factor receptor 2 gene (HER2) [4, 5]. HER2 + tumors are characterized by aggressive tumor growth and are associated with an increased risk of recurrence and metastasis [6–8]. The introduction of HER2-targeted therapies such as trastuzumab, pertuzumab, and trastuzumab emtansine (HER2 targeted antibody drug conjugates) has significantly improved clinical outcomes in both early-stage and advanced disease [9–11]. However, in the metastatic setting of breast-to-brain disease, many patients develop recurrence with limited treatment options, due to the selectivity of the blood brain barrier [8, 12, 13]. As a result, metastatic HER2 + breast cancer remains a clinical challenge, underscoring the need for strategies that better characterize therapeutic response.

Tucatinib is an orally bioavailable tyrosine kinase inhibitor that selectively targets HER2 while minimizing off-target inhibition of EGFR [14]. This increased specificity reduces gastrointestinal and dermatologic toxicities commonly observed with pan-HER inhibitors [15]. Clinical studies, including the HER2CLIMB trial, have demonstrated that tucatinib in combination with trastuzumab and capecitabine improves overall and progression-free survival in patients with metastatic HER2 + breast cancer, including those with active brain metastases [16–18]. While tucatinib’s clinical efficacy has been well established, its biological effects on tumor cell growth and associated tumor microenvironment (TME) characteristics, including changes in perfusion, hypoxia, proliferation, and HER2 expression, remain relatively unexplored. As these are key factors in understanding variability in response and personalization therapy decision making, characterizing how targeted HER2 inhibition with tucatinib alters the TME could enable improved patient stratification and treatment monitoring through non-invasive imaging biomarkers.

Molecular imaging enables noninvasive assessment of cellular and molecular changes during therapy, with positron emission tomography (PET) serving as a robust and quantitative platform to track tumor biology in vivo. Traditional anatomical imaging approaches fail to capture the molecular alterations that precede changes in tumor size, limiting their utility in understanding biological alterations that affect therapy response. While [^18^F]FDG-PET is the clinical standard for evaluating glucose metabolism, other advanced tracers can provide more specific biological information about TME dynamics and therapy response in breast cancer. For instance, [^18^F]fluoromisonidazole (FMISO) measures tumor hypoxia—a critical driver of immune evasion and drug resistance—whereas [^18^F]fluorothymidine (FLT) reflects thymidine kinase 1 activity, serving as a surrogate for active cell proliferation [4, 5, 19, 20]. In HER2 + breast cancer models, Whisenant et al. demonstrated that FLT-PET detects early proliferative responses to trastuzumab in trastuzumab-sensitive xenografts, preceding volumetric changes, and supports no changes in imaging metrics in resistant tumors [21]. Similarly, Mansur et al. found that histogram-based FLT-PET analysis successfully captured intratumoral heterogeneity and early treatment effects during trastuzumab and PARP inhibitor (niraparib) combination therapy in patient derived xenograft models [4]. [^89^Zr]Zr-pertuzumab PET complements these approaches by visualizing HER2 receptor availability and internalization in response to targeted agents like tucatinib. Massicano et. al demonstrated that [^89^Zr]Zr-pertuzumab PET could delineate response to anti-HER2 antibody drug conjugate, Trastuzumab Emtansine [22]. Collectively, these molecular imaging strategies provide a multidimensional understanding of therapy-induced tumor biology and TME changes, enabling dynamic response assessment beyond conventional tumor size changes.

Despite the clinical success of tucatinib, there remains a need to understand how selective small molecule inhibitors influence the TME in HER2 + breast cancer to best determine how to stratify patients and integrate additional therapies. In this study, we employed a PET imaging approach to non-invasively characterize how HER2-targeted tucatinib therapy remodels the TME in preclinical models of HER2 + breast cancer. By integrating [^18^F]-FLT, [^18^F]-FMISO, and [^89^Zr]Zr-pertuzumab PET imaging during tucatinib therapy, we aimed to quantify changes in tumor proliferation, hypoxia, and HER2 expression, respectively, in both cell line–derived and patient-derived xenograft models, which better recapitulate clinical cancer due to their increased heterogeneity. Through this comprehensive imaging strategy, we sought to define the temporal dynamics of tucatinib response, identify potential biomarkers for therapeutic monitoring, and lay the foundation for future translation of molecular imaging into HER2-directed treatment paradigms with tucatinib. As the landscape for therapeutics in HER2 + breast cancer continue to evolve and broaden, there remains a clinical need for noninvasive imaging biomarkers that can capture early biological response to HER2-targeted therapy non-invasively and help identify which therapeutics may be most beneficial on a personalized basis. While anatomical imaging with MRI provides critical information on tumor size alone, molecular imaging may provide a unique characterization of tumors during therapy to understand biological changes prior to downstream changes in tumor size. Molecular imaging approaches capable of longitudinally assessing proliferation, hypoxia and HER2 may provide complementary metrics to conventional imaging by identifying treatment induced changes in the tumor microenvironment which may provide timing of when to switch therapies or add a secondary treatment.

## Materials and methods

### Cell culture and tumor models

The HER2 + cell line, BT474, was acquired from ATCC and was maintained in Improved Modified Eagle’s Media (IMEM) supplemented with 10% Fetal Bovine Serum FBS and 0.5% Insulin. Cell lines tested negative for pathogens through Charles River Research Animal Diagnostic Services. All animal procedures were performed in accordance with The University of Alabama at Birmingham’s Institutional Animal Care and Use Committee under APN 21611. Five- to six-week-old female athymic nude mice were obtained from Charles River Laboratories (Catalog number: 490). To establish subcutaneous models, 1 × 10^7^ BT474 cells were engrafted with 30% Matrigel and 70% serum free IMEM on the subcutaneous right hind flank.

For patient derived xenograft (PDX) models, six-week-old female NOD.Cg-Prkdc^scid^Il2rg^tm1Wjl^ISzJ (NSG) mice were obtained from Jackson Labs (catalog number: 005557) and subcutaneously implanted with 0.72 mg 17β-estradiol pellets under 2% isofluorane anesthesia. Approximately 24 h later, 100–150 mm^3^ tumor pieces of BCM 3472 (HER2 +) were surgically engrafted into the cleared third mammary fat pad with Matrigel supplement under 2% isofluorane anesthesia [23].

To monitor longitudinal molecular changes during tucatinib therapy, mice were imaged with PET imaging (FLT-PET, FMISO-PET or [^89^Zr]Zr-pertuzumab PET) on day 0 (baseline) and immediately began daily tucatinib therapy. Mice were treated with 50 mg/kg tucatinib via oral gavage daily until the final imaging timepoint. Following imaging, tumors were excised and sectioned for immunohistochemistry (IHC).

### FLT-PET imaging to monitor changes in tumor proliferation in response to tucatinib therapy

To determine changes in tumor proliferation in tucatinib-treated HER2 + breast cancers, HER2 + cell line derived tumors (BT474, N = 8 per condition) or PDX tumors (BCM3472, N = 4 per condition) were imaged with FLT-PET imaging during therapy. [^18^F]-FLT was synthesized by the University of Alabama at Birmingham cyclotron facility on a GE FASTlab2 synthesizer, according to literature procedures [4, 24, 25]. Mice were imaged on days 0, 3, and 7. Briefly, mice were retro-orbitally injected with approximately 100 μCi of [^18^F]-FLT and, 60 min later, were imaged with a preclinical PET/CT scanner (Sofie Biosciences, Somerset, NJ, USA) with a 20 min F-18 static PET scan and an 80 kVp bin 2 CT. Regions of interest (ROI) were drawn on the CT images to identify the tumor and overlaid onto PET images to calculate the mean standard uptake value (SUV). SUVmean was defined as the average activity concentration within a ROI normalized to injected dose and body weight [26] and quantified with the following formula:

$$SUV = \frac{radioactive concentration}{{injected activty/body weight}}$$

Tumor ROIs were manually drawn on a slice-by-slice manner using the CT for anatomical reference. For tumor-to-muscle ratio, a reference muscle ROI was placed on the contralateral hindlimb muscle. The CT and PET data are automatically co-registered, as the mouse is stationary between scan acquisitions, therefore the ROIs from the CT images are utilized for PET quantification. [^18^F]-FLT acquisition revealed a larger range of variability of systemic tracer distribution, therefore the tracer was normalized to the muscle SUV (which has been shown to have minimal active proliferation and can serve as a reference tissue [27] to calculate the tumor-to-muscle SUV ratio.

### FMISO-PET imaging to monitor changes in tumor hypoxia in response to tucatinib therapy

To determine changes in tumor hypoxia in tucatinib treated HER2 + breast cancers, HER2 + cell line derived tumors (BT474, N = 4–5 per condition) or PDX tumors (BCM3472, N = 5–9 per condition) were imaged with FMISO-PET during therapy on days 0, 3, and 7. [^18^F]-FMISO was synthesized by the University of Alabama at Birmingham cyclotron facility on a GE FASTlab2 synthesizer, according to literature procedures [28, 29]. Briefly, mice were retro-orbitally injected with approximately 150 μCi of [^18^F]-FMISO and, 80 min later, were imaged followed by analysis as previously described. Tumor hypoxia was quantified using SUV_mean_ to reflect average tracer uptake across the entire tumor volume, consistent with prior preclinical FMISO-PET studies evaluating overall hypoxia [5, 30–32].

### [^89^Zr]Zr-Pertuzumab imaging to monitor changes in tumor HER2 expression in response to tucatinib therapy

To determine changes in tumor HER2 expression in tucatinib treated HER2 + breast cancers, HER2 + cell line derived tumors (BT474, N = 5 per condition) or PDX tumors (BCM3472, N = 5 per condition) were imaged with [^89^Zr]Zr-Pertuzumab-PET imaging on day 0 and 14. [^89^Zr]Zr-oxalate was purchased as a service by The University of Alabama at Birmingham’s cyclotron facility. Pertuzumab (Medchemexpress, Catalog #: HY-P9912) was conjugated with 0.4 mg of Deferoxamine (DFO) at a concentration of 10 mg/mL rocking at room temperature overnight. DFO-Pertuzumab was labeled with [^89^Zr]Zr-oxalate at a specific activity of 10 μCi/μg [19]. Following radiochemistry, tumor bearing mice were injected with approximately 75 μCi of [^89^Zr]Zr-Pertuzumab seven days prior to their imaging time points. Mice were imaged with a static 20 min PET followed by a 5 min CT to identify changes in HER2 expression following tucatinib therapy. SUV analysis was performed as previously described. HER2 expression was quantified using SUV_mean_ to reflect average tracer uptake across the entire tumor volume, consistent with prior preclinical [^89^Zr]Zr-Pertuzumab studies evaluating overall HER2 expression [19, 20, 22].

### Immunohistochemistry

Following final imaging timepoint, tumors were excised for IHC to biologically validate molecular PET imaging. Tumors were fixed in 10% neutral buffered formalin and paraffin embedded before being sectioned to 5-micron slices. Each slice was stained with 1:300 Ki-67 (Abcam, Catalog #: ab16667), 1:100 HIF-1α (Thermofisher Scientific, Catalog #: 14–9100-82), and 1:100 HER2 (Cell Signaling Technologies, Catalog #: 2242) overnight in 1% BSA in TBST. Following primary antibody staining, slides were counterstained using a secondary antibody mouse/rabbit IgG VisUCyte HRP Polymer Antibody (R&D Systems, Catalog #: VC002-050) for approximately 1 h. Following secondary staining, slides were developed with HRP DAB Substrate Kit (Vector Laboratories, Catalog: SK-4100) and stained with hematoxylin. Following staining, sections were scanned with an EVOS M7000 imaging system (ThermoFisher, Waltham, MA, USA). Nonviable tumor regions were removed from quantitative IHC analysis.

### Quantification of immunohistochemistry staining

Quantification of histological stains was performed using a standardized QuPath (v. 0.5.1) approach [33]. Initially, tissue was segmented using optimal thresholding, followed by manual corrections to remove necrotic and non-tumor regions, as defined by H&E staining. Ki-67 stain cell positivity was determined using a standardized threshold across all samples, with percent positivity being defined as total Ki-67 positive cells divided by all cells. For HIF-1α and HER2, quantification was performed on a regional basis as defined by a standardized threshold across all samples, with percent positive being defined as total positively stained area divided by total tumor area.

### Statistical analysis

Experimental conditions were summarized by average of replicates and error was represented by standard error of mean (SEM). Significance between experimental conditions was assessed using a Mann–Whitney U Test. All data and figures were analyzed and generated using GraphPad Prism 7 (La Jolla, CA, USA).

## Results

### *Baseline tumor microenvironment changes and tucatinib response differs between cell line and PDX models of HER2* + *breast cancer*

**Fig. 1:**
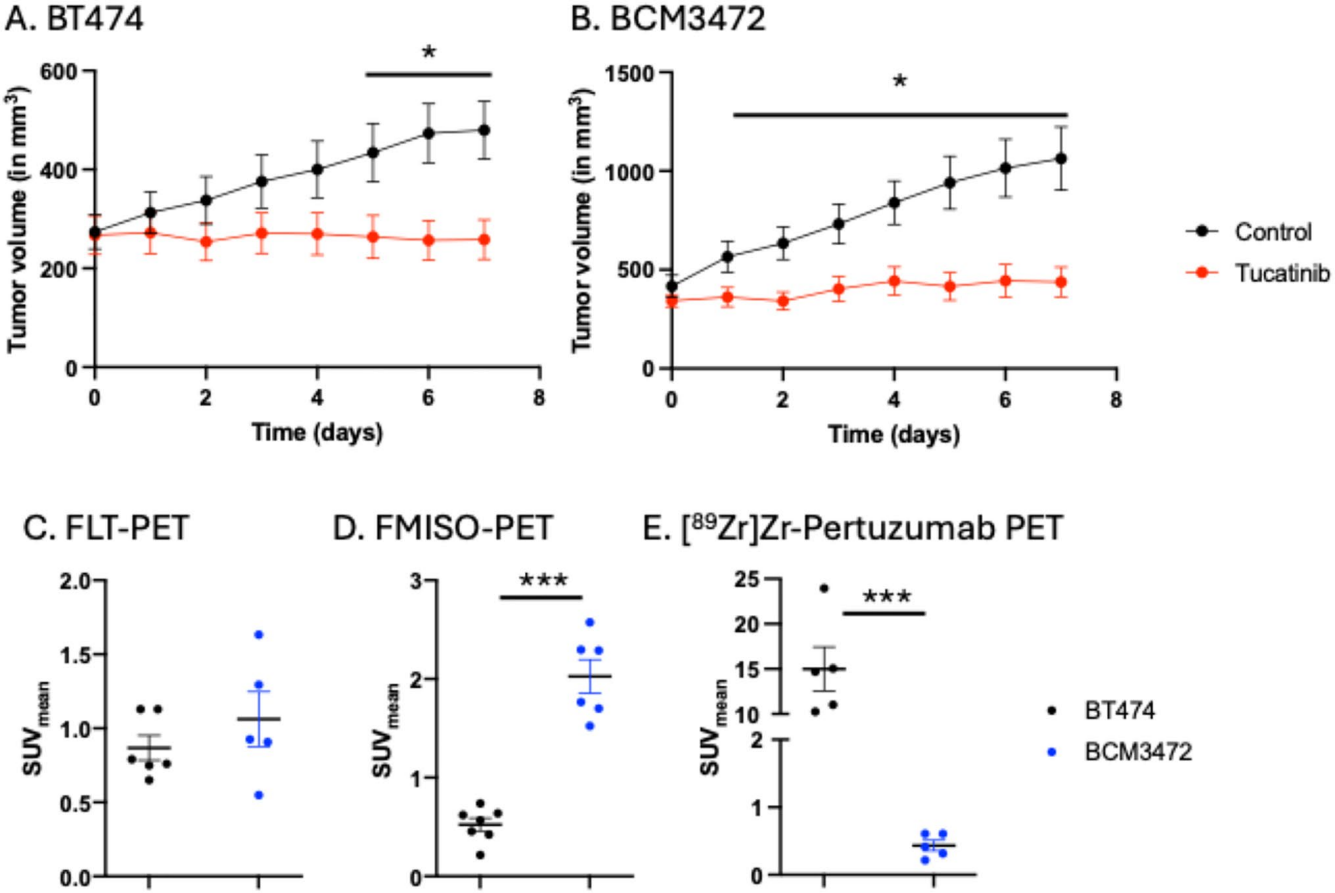
Determining response to tucatinib in a cell line derived and patient derived model of HER2 + breast cancer. BT474 (**A**) and BCM3472 (**B**) tumors were treated with tucatinib daily (50 mg/kg via oral gavage). FLT-PET reveals no significant changes in tumor proliferation between BT474 and BCM3472 tumor models (**C**). FMISO PET (**D**) and [^89^Zr]Zr-Pertuzumab PET reveal significant alterations in baseline hypoxia and HER2 expression, respectively

To examine how tucatinib response differs in cell line derived models of HER2 + breast cancer and PDX models of HER2 + breast cancer, cell line derived HER2 + BT474 tumor models and HER2 + PDX, BCM3472 tumor models, were treated with tucatinib daily and monitored for long term changes in tumor volume. For both BT474 and PDX tumors, there were no significant differences in tumor volume at baseline. BT474 tumors treated with tucatinib therapy showed significant decreases in tumor volume beginning on day 5 (Control: 479.5 ± 248.8 mm^3^, tucatinib: 258.4 ± 187.5 mm^3^, p < 0.01) (Fig. 1A). PDX tumors treated with tucatinib therapy showed significant decreases in tumor volume beginning on day 1 (Control: 1063.7 ± 661.1 mm^3^, tucatinib: 437.7 ± 290.9 mm^3^, p < 0.01) (Fig. 1B). At baseline, BT474 and PDX tumors showed no difference in proliferation, as quantified through FLT-PET (0.9 ± 0.2 SUV vs 1.1 ± 0.4 SUV, respectively, p = 0.34) (Fig. 1C). In contrast, at baseline, HER2 + PDX tumors showed significantly heightened hypoxia compared to HER2 + BT474 tumors, as quantified through FMISO-PET (Fig. 1D). Further, as expected the PDX tumors demonstrated significantly lower HER2 expression, quantified through [^89^Zr]Zr-Pertuzumab PET, relative to the cell line derived HER2 + BT474 tumors (p < 0.01) (Fig. 1E).

### *Tucatinib significantly reduces tumor proliferation in preclinical models of HER2* + *breast cancer*

**Fig. 2:**
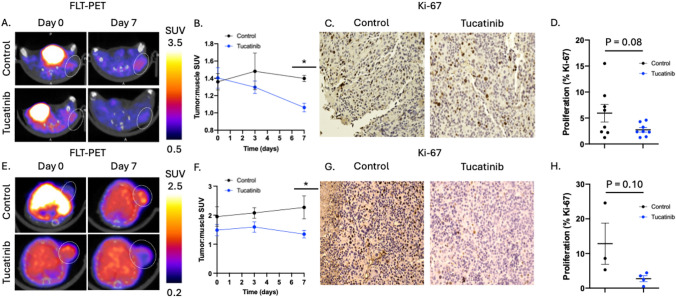
[.^18^F]-FLT PET imaging identifies tucatinib driven changes in tumor proliferation. BT474 tumors were treated with tucatinib and imaged with proliferation FLT-PET imaging (**A**). FLT-PET reveals significant decreases in proliferation by day 7 (**C**). Tumors were excised following imaging and stained for Ki-67 to biologically validate changes in proliferation with quantitative IHC analysis (**D**, **E**). Results were validated with HER2 + PDX BCM3472 (F–J)

To examine how tucatinib affects HER2 + breast cancer tumor proliferation, [^18^F]-FLT PET imaging was used to study longitudinal changes in tumor proliferation in response to anti-HER2 tucatinib therapy (Fig. 2A). BT474 tumors treated with tucatinib had significantly lower proliferation (tumor:muscle SUV) compared to control on day 7 (Control: 1.40 ± 0.09 tumor:muscle SUV, tucatinib-treated: 1.06 ± 0.12 tumor:muscle SUV, p < 0.01) (Fig. 2B). Changes in tumor proliferation were validated through Ki-67 IHC (Fig. 2C). BT474 control tumors had 5.94 ± 4.79% Ki-67 positivity, while BT474 tumors treated with daily tucatinib had 2.72 ± 1.23% Ki-67 positivity (*p* = 0.08) (Fig. 2D). Tucatinib driven changes in tumor proliferation were also confirmed in a HER2 + PDX tumor model (Fig. 2E-2H). HER2 + PDX tumors treated with tucatinib had significantly lower tumor:muscle SUV on day 7 (Control: 2.27 ± 0.88 tumor:muscle SUV, tucatinib: 1.35 ± 0.32 tumor:muscle SUV, p < 0.05) (Fig. 2E, 2F). Changes in tumor proliferation were validated through Ki-67 IHC (Fig. 2G). PDX control tumors treated revealed 12.8 ± 10.3% Ki-67 positivity, while PDX tumors treated with daily tucatinib had 2.7 ± 1.7% Ki-67 positivity (*p* = 0.10) (Fig. 2H).

### *Tucatinib significantly reduces tumor hypoxia in preclinical models of HER2* + *breast cancer*

**Fig. 3:**
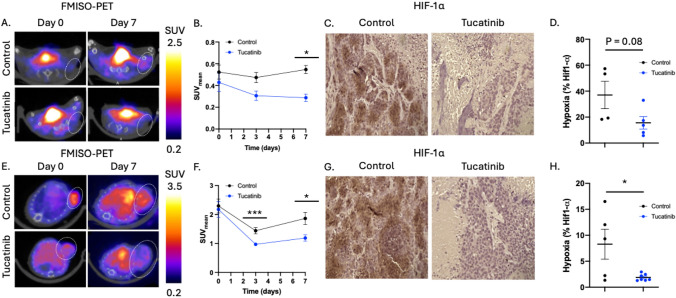
[^18^F]-FMISO PET imaging identifies tucatinib-driven changes in tumor hypoxia. BT474 tumors were treated with tucatinib and imaged with hypoxia-guided FMISO-PET imaging (Fig. 3A). FMISO-PET shows significant reductions in hypoxia by day 7, as measured by FMISO uptake (C). Tumors were excised following imaging and stained for HIF-1α to biologically validate changes in hypoxia with quantitative IHC analysis (D, E). Results were validated in the HER2 + PDX model BCM3472 (F–J)

To examine how tucatinib affects HER2 + breast cancer tumor hypoxia, [^18^F]-FMISO PET imaging was used to study longitudinal changes in tumor hypoxia in response to anti-HER2 tucatinib therapy (Fig. 3A). BT474 tumors treated with tucatinib had significantly lower SUV_mean_ on day 7 (Control: 0.62 ± 0.05 SUV, tucatinib: 0.29 ± 0.09 SUV, p < 0.01) (Fig. 3B). Changes in tumor hypoxia were validated through HIF-1α IHC (Fig. 3C). BT474 control tumors had 37.04 ± 21.1% HIF-1α positivity, while BT474 tumors treated with tucatinib had 15.6 ± 10.7% HIF-1α positivity (p = 0.08) (Fig. 3D). Tucatinib driven changes in tumor hypoxia were also replicated in the HER2 + PDX tumor model (Fig. 3E-3H). PDX tumors treated with tucatinib had significantly lower SUV_mean_ on day 7 (Control: 1.86 ± 0.51 SUV, tucatinib: 1.19 ± 0.22 SUV, p < 0.01) (Fig. 3E, 3F). Changes in tumor hypoxia were validated through HIF-1α IHC (Fig. 3G). PDX control tumors had 8.29 ± 6.47% HIF-1α positivity, while tucatinib-treated had 1.89 ± 0.65% HIF-1α positivity (p = 0.02) (Fig. 3H).

### *Tucatinib significantly reduces tumor HER2 expression in preclinical models of HER2* + *breast cancer*

**Fig. 4:**
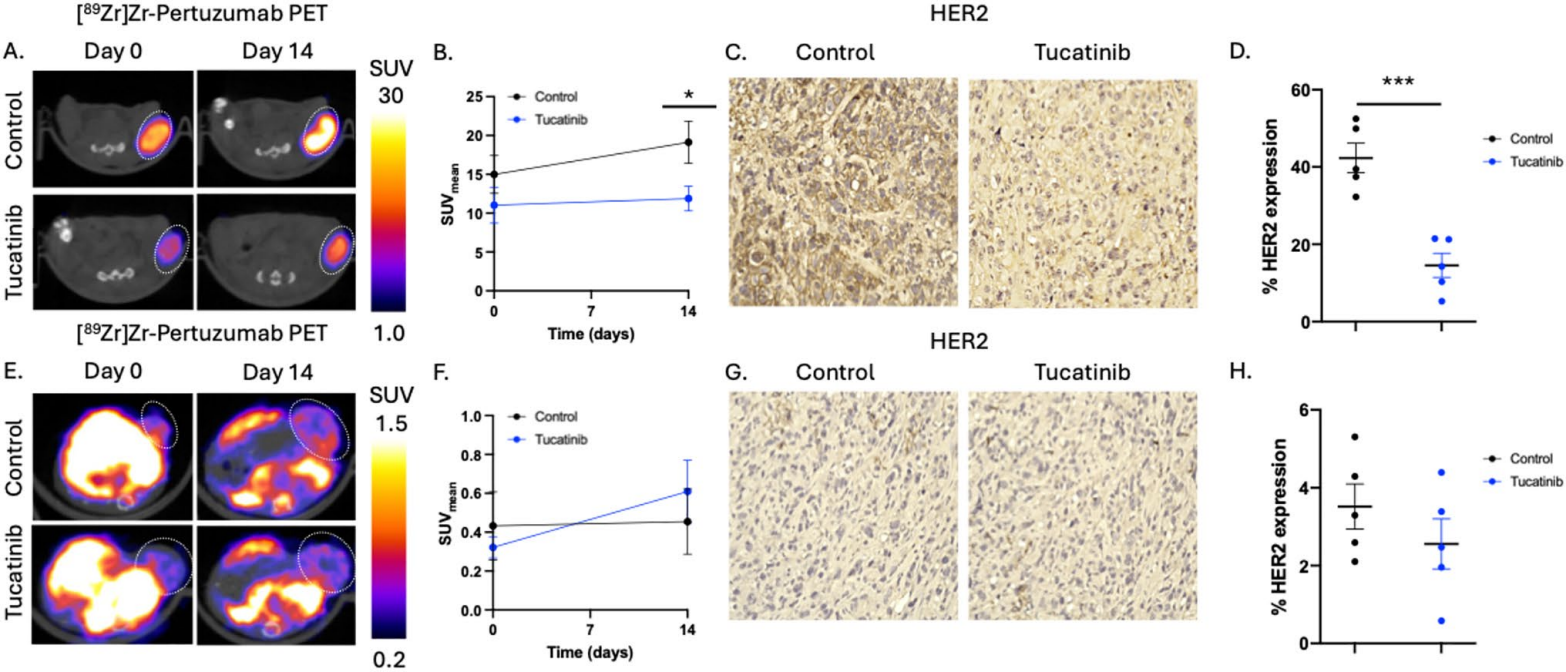
[^89^Zr]Zr-Pertuzumab PET imaging identifies tumor model dependent changes in HER2 expression following tucatinib therapy. BT474 tumors were treated with tucatinib and imaged with HER2-targeted [^89^Zr]Zr-pertuzumab PET imaging (A). [^89^Zr]-Pertuzumab PET reveals significant reductions in HER2 expression by day 14, as measured by PET SUVmean (C). Tumors were excised following imaging and stained for HER2 to biologically validate changes in receptor expression with quantitative IHC analysis (D, E). In contrast, HER2 + PDX BCM3472 tumors did not demonstrate significant changes in [^89^Zr]Zr-pertuzumab PET uptake or HER2 IHC following tucatinib treatment, despite reductions in tumor volume, indicating HER2 + model-specific differences in HER2 modulation (F–J)

To examine how tucatinib affects HER2 expression, [^89^Zr]Zr-Pertuzumab PET imaging was used to study longitudinal changes in tumor HER2 expression in response to anti-HER2 tucatinib therapy (Fig. 4A). BT474 tumors treated with tucatinib observed significant decreases in SUV_mean_ on day 7 (Control: 19.1 ± 6.1 SUV, tucatinib: 11.9 ± 3.6 SUV, p < 0.05) (Fig. 4B). Changes in tumor HER2 expression were validated through HER2 IHC (Fig. 4C). BT474 tumors treated with a control had 42.3 ± 8.6% HER2 positivity, while BT474 tumors treated with daily tucatinib had 14.5 ± 7.0% HER2 positivity (p < 0.01) (Fig. 4D). Conversely, this was not replicated in PDX tumor model (Fig. 4E-4H), as tucatinib treated PDX tumors HER2 expression was not significantly different between cohorts based on imaging (Fig. 4E, 4F). Changes in tumor HER2 expression through IHC also revealed no significant alterations in HER2 expression via IHC (3.5 ± 1.3% vs 2.6 ± 1.4% HER2 positivity, *p* = 0.30) (Fig. 4 G,H).

## Discussion

In this study, we quantified and compared therapy induced biological changes via non-invasive PET imaging strategies to characterize response to HER2-targeted tucatinib therapy in preclinical human cell line and PDX models of HER2 + breast cancer. Using [^18^F]-FLT, [^18^F]-FMISO, and [^89^Zr]Zr-pertuzumab PET imaging, we evaluated therapy induced biological changes encompassing both tumor cell properties (proliferation and HER2 receptor availability) and tumor microenvironment changes (hypoxia). Clinically, these findings highlight the utility of PET imaging as a noninvasive strategy to monitor important components of the tumor microenvironment that impact therapy, including cell proliferation, oxygenation and the status of HER2. Early biological responses to tucatinib are seen beyond conventional size-based assessments and importantly, these imaging-derived changes may also inform on variations of response across the heterogeneous tumor (e.g. some regions decreasing in proliferation while others not during the therapy [4, 34] reflects variations in therapeutic response) or increased susceptibility to combination therapies as (e.g. reduction in tumor hypoxia detected by FMISO-PET may reflect increased radiosensitivity or sensitivity to chemotherapy [5, 31, 35–37]). Understanding the kinetics and biological impact that advanced imaging can capture, can help to tailor treatment sequencing and timing to have the greatest impact on the tumor. Tucatinib has emerged as a critical agent in the treatment landscape of metastatic HER2 + breast cancer, particularly following the success of clinical trials, which demonstrated improved overall survival and intracranial response rates when tucatinib was combined with trastuzumab and capecitabine [14, 16]. Subsequent studies have explored its activity in models with brain metastases, reinforcing its ability to cross the blood–brain barrier and target CNS lesions, and have demonstrated that tucatinib synergizes with immunotherapy [38, 39]. Despite these advances, the biological effects of tucatinib on the tumor microenvironment are not completely understood, especially in terms of how HER2 inhibition influences hypoxia, cellular proliferation, and receptor modulation at the tumor site. Our data demonstrates that tucatinib monotherapy significantly reduced tumor volume (as has been previously demonstrated preclinically and clinically [40–42]), proliferation, and hypoxia across both cell line–derived (BT474) and patient-derived xenograft (BCM3472) models. Furthermore, we observed model-dependent differences in HER2 modulation, with [^89^Zr]Zr-pertuzumab PET and HER2 IHC revealing significant downregulation of HER2 in BT474 tumors but not in BCM3472 tumors that could be derived from baseline differences in HER2 expression. Variations in PET metrics of response were expected as there is differential baseline HER2 expression between the BT474 cell line based model and the BCM3472 PDX based model. BT474 tumors are characterized by relatively homogenous and high HER2 expression [43], which may cause HER2-high cells to exhibit increased susceptibility to tucatinib. This preferential killing of HER2-high cells causes an overall reduction in measured HER2 expression [44, 45]. Whereas, BCM3472 PDX, while known to retain clinical heterogeneity, was extracted from a HER2 lower expressing tumor [46] and did not observe the same decrease in overall HER2 expression post tucatinib therapy. These findings suggest that PET imaging can inform on heterogeneity in therapeutic response and provide mechanistic insight into how HER2 targeted small molecule inhibitors alter tumor biology and associated microenvironmental features.

The imaging changes that were found after tucatinib therapy may reflect a shift in tumor biology toward a less aggressive phenotype and could sensitize tumors to subsequent therapies, including radiation or immune-based strategies [5, 30, 32, 43]. Hypoxia is a known driver of treatment resistance, angiogenesis, and immune evasion in solid tumors, and its reduction may enhance drug delivery and immune infiltration [5, 30, 32, 43]. Similarly, decreases in [^18^F]-FLT uptake suggest that tucatinib disrupts tumor cell cycle progression, consistent with HER2’s role in cell and mitogenic signaling [47–49]. These findings support the concept that HER2 inhibition has effects beyond direct tumor cell killing, potentially reshaping the tumor microenvironment in ways that favor reduced tumor burden. The effects of antibody-based anti-HER2 inhibitors, like trastuzumab, have been studied on the TME in preclinical HER2 + breast cancer models. Trastuzumab treatment has been shown to significantly reduce tumor hypoxia in BT474 xenografts, with changes confirmed by pimonidazole immunohistochemistry, suggesting a favorable shift in the TME that could enhance therapeutic sensitivity [5, 50]. Building on this, it was shown that trastuzumab-induced reductions in hypoxia were sufficient to achieve radiosensitization and enhance chemotherapy effectiveness in HER2 + tumors, underscoring the interplay between HER2 signaling, oxygenation, and radiation sensitivity [5]. Complementary to these findings, it has been shown that FLT-PET imaging can be used to predict early responses to trastuzumab in sensitive HER2 + models, demonstrating that changes in proliferation could be detected prior to tumor volume reductions [5, 21]. These tucatinib-driven decreases in proliferation and hypoxia mirror the molecular and microenvironmental effects that has been observed with trastuzumab, suggesting similar downstream mechanisms despite the pharmacological differences between antibodies and small molecule inhibitors. Collectively, these studies highlight the value of advanced imaging biomarkers in capturing early, mechanistic effects of HER2-targeted therapies, insights that parallel our observations with tucatinib.

The limitations of this study stem from the fact that modeling human HER2 + breast cancer requires the use of immunodeficient mice, which do not capture immune mediated effects. Similarly, HER2 therapies have been shown to modulate vascular perfusion and immune cell populations; therefore these elements and their relationship with tucatinib should be explored in the future. Overall, these findings highlight the utility of PET imaging to non-invasively quantify the dynamic effects of targeted therapy on tumor biology. By capturing early changes in proliferation, hypoxia, and receptor expression, molecular imaging provides a more nuanced assessment of therapeutic response than tumor volume alone. While our study was limited to subcutaneous xenograft models in immunodeficient mice, the observed changes demonstrate tucatinib-induced biological changes that may be relevant to combination therapy strategies, warranting further investigation in more clinically representative models. Future studies should explore these imaging biomarkers in metastatic breast cancer models and in the context of immunocompetent systems to better reflect clinical scenarios. Ultimately, these findings support the potential role of molecular imaging as a translational research tool to characterize early biological response to HER2-targeted therapy, with future validation required before application to clinical decision-making or personalized treatment strategies.

## Data Availability

Datasets and materials are available upon reasonable request.
